# CCAR2 negatively regulates IL-8 production in cervical cancer cells

**DOI:** 10.18632/oncotarget.23199

**Published:** 2017-12-13

**Authors:** Wootae Kim, Jaehyuk Pyo, Byeong-Joo Noh, Joo-Won Jeong, Juhie Lee, Ja-Eun Kim

**Affiliations:** ^1^ Department of Biomedical Science, Graduate School, Kyung Hee University, Seoul 02447, Republic of Korea; ^2^ Department of Pathology, School of Medicine, Kyung Hee University, Seoul 02447, Republic of Korea; ^3^ Department of Anatomy and Neurobiology, School of Medicine, Kyung Hee University, Seoul 02447, Republic of Korea; ^4^ Department of Pharmacology, School of Medicine, Kyung Hee University, Seoul 02447, Republic of Korea

**Keywords:** CCAR2, IL-8, oxidative stress, cervical cancer

## Abstract

Cell cycle and apoptosis regulator 2 (CCAR2) is a multifaceted protein that controls diverse cellular functions; however, its function in cancer is unclear. To better understand its potential role in cancer, we examined gene expression patterns regulated by CCAR2 in cervical cancer cells. Cytokine and chemokine production by CCAR2-deficient cells increased under oxidative conditions. In particular, H_2_O_2_-treated CCAR2-depleted cells showed a significant increase in interleukin-8 (IL-8) production, indicating a negative regulation of IL-8 by CCAR2. Upregulation of IL-8 expression in CCAR2-deficient cells occurred via activation of transcription factor AP-1. The negative correlation between CCAR2 and IL-8 expression was confirmed by examining mRNA and protein levels in tissues from cervical cancer patients. Furthermore, CCAR2-regulated IL-8 expression is associated with a shorter survival of cervical cancer patients. Overall, the data suggest that CCAR2 plays a critical role in controlling both the cancer secretome and cancer progression.

## INTRODUCTION

Cell cycle and apoptosis regulator 2 (CCAR2, formerly known as DBC1 [deleted in breast cancer 1]) is a multifaceted protein that regulates a diverse subset of cellular functions. CCAR2 controls transcription, mRNA splicing, DNA damage responses, circadian rhythm, inflammation, metabolism, differentiation, proliferation, survival, and apoptosis [1–3]. With respect to cancer growth, CCAR2 is thought to act as a tumor promoter or tumor suppressor depending on the context.

The role of CCAR2 as a tumor promoter is supported by findings that it promotes proliferation [4–12], migration, and invasion of cancer cells [6, 8, 9, 13] and suppresses cancer cell anoikis [14]. Depleting CCAR2 inhibits the growth of tumor xenografts derived from colon cancer [8] and laryngeal squamous cell carcinoma cells [5]. By contrast, CCAR2 functions as a tumor suppressor by stabilizing p53 through competitive inhibition of Mdm2 binding [15]. CCAR2 knockout mice spontaneously develop tumors of the liver and lung, teratomas, and lymphomas [15]. CCAR2 knockout mouse embryonic fibroblasts proliferate faster than wild-type cells [15], which is the opposite phenotype to that observed in cancer cell lines. Therefore, although the exact role of CCAR2 as a tumor promoter or tumor suppressor in humans is unclear, CCAR2 plays an intimate part in tumor development and progression.

Recently, the tumor microenvironment has emerged as a critical niche that regulates tumor progression, angiogenesis, and metastasis [16]. Beyond tumor cells, the microenvironment comprises diverse types of cells, secreted soluble factors (e.g., cytokines and growth factors), and the extracellular matrix [17]. Within this heterogeneous tumor microenvironment, tumor cells, endothelial cells, cancer-associated fibroblasts, and infiltrating inflammatory cells produce many different cytokines that control local networks [18]. Cytokines have pro-tumorigenic or anti-tumorigenic effects [19]. Among them, interleukin-8 (IL-8), known as CXCL8 (C-X-C motif chemokine ligand 8), is a pro-inflammatory chemokine [20] that binds CXCR1 and CXCR2 (C-X-C chemokine receptor type 1 and 2), both of which are cell-surface G protein-coupled receptors [21]. The autocrine and paracrine functions of IL-8 activate the intracellular signaling and mediate pro-tumorigenic effects, including epithelial-mesenchymal transition, survival, proliferation, migration, invasion, angiogenesis and resistance to anoikis [22–26]. In addition, the complex tumor microenvironment is oxidative in nature. Reactive oxygen species (ROS) produced by tumor cells, cancer-associated fibroblasts, cancer-associated macrophages, and senescent fibroblasts create a sustained pro-inflammatory environment, again by inducing secretion of diverse cytokines [27]. Finally, ROS-induced inflammation accelerates malignancy by increasing cancer cell survival, proliferation, invasion, angiogenesis, and metastasis [28, 29].

Although the link between CCAR2 and tumor cells has been studied, the role of CCAR2 in the tumor microenvironment remains unclear. In addition, although the previous studies show that CCAR2 regulates tumor cell proliferation, migration, and invasion, the mediators of CCAR2 functions have not been identified. Here, we show that CCAR2 modulates expression and secretion of IL-8 by tumor cells under oxidative conditions. Thus, CCAR2 is a critical factor that fine-tunes the tumor microenvironment.

## RESULTS

### CCAR2 regulates the expression of cytokine and chemokine following oxidative stress

**Figure 1:**
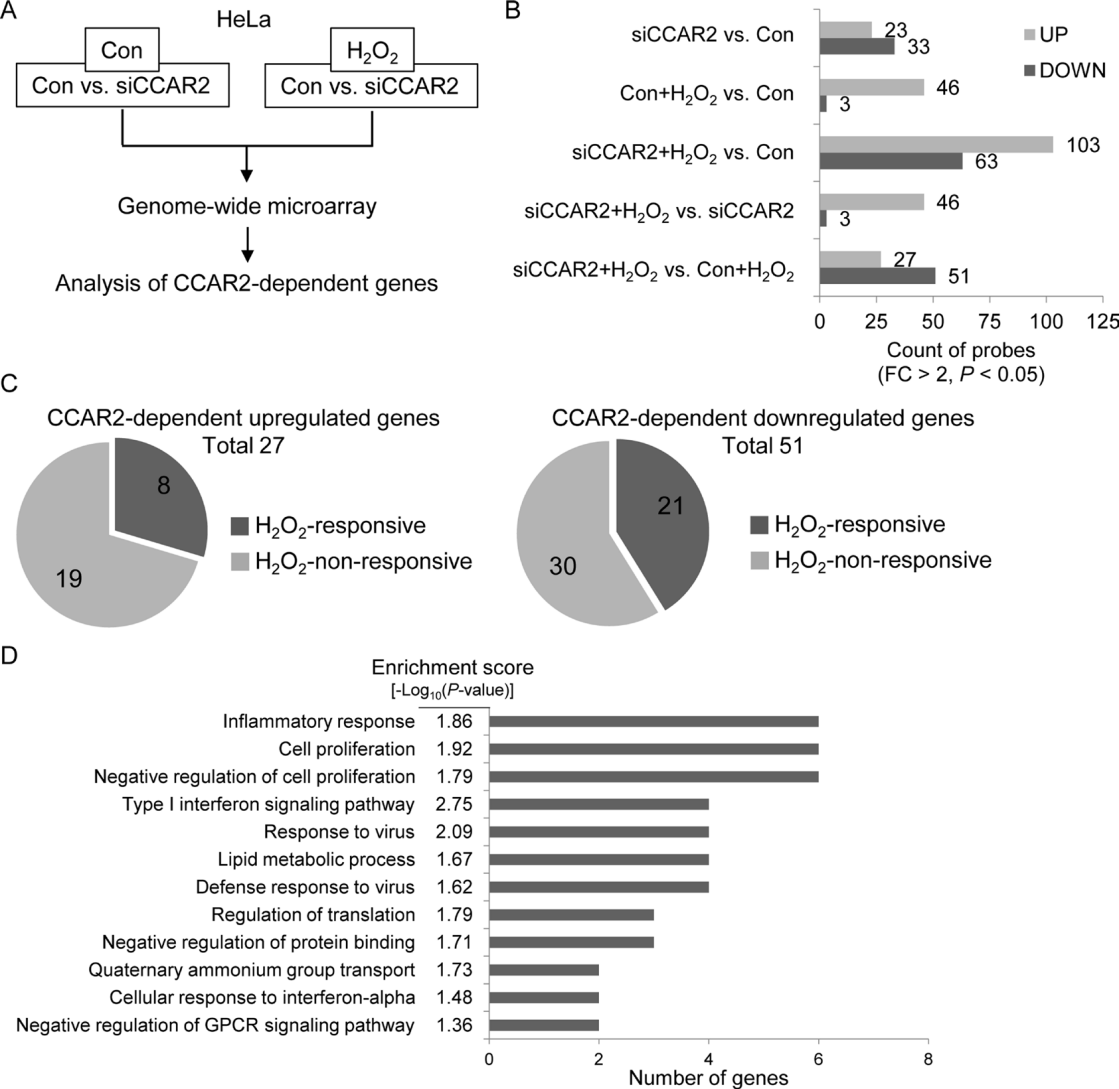
CCAR2 affects gene expression in response to oxidative stress HeLa cells were transfected with siCCAR2 (siC). Forty-eight hours later, cells were treated with 0.5 mM H_2_O_2_ for 2 h. (**A**) Microarray was performed and the results were analyzed. (**B**, **C**) Only genes showing a FC > 2 and a *P* value < 0.05 were counted. (B) Bar graph shows the number of up- and downregulated genes in the indicated groups. (C) Pie graph shows the number of H_2_O_2_-responsive genes dependent on CCAR2 for efficient up- (left) or downregulation (right). (**D**) GO term analysis was performed to identify biological processes. The annotation cluster is shown according to number of genes with a cluster enrichment score [-log_10_(*P*-value)].

**Figure 2:**
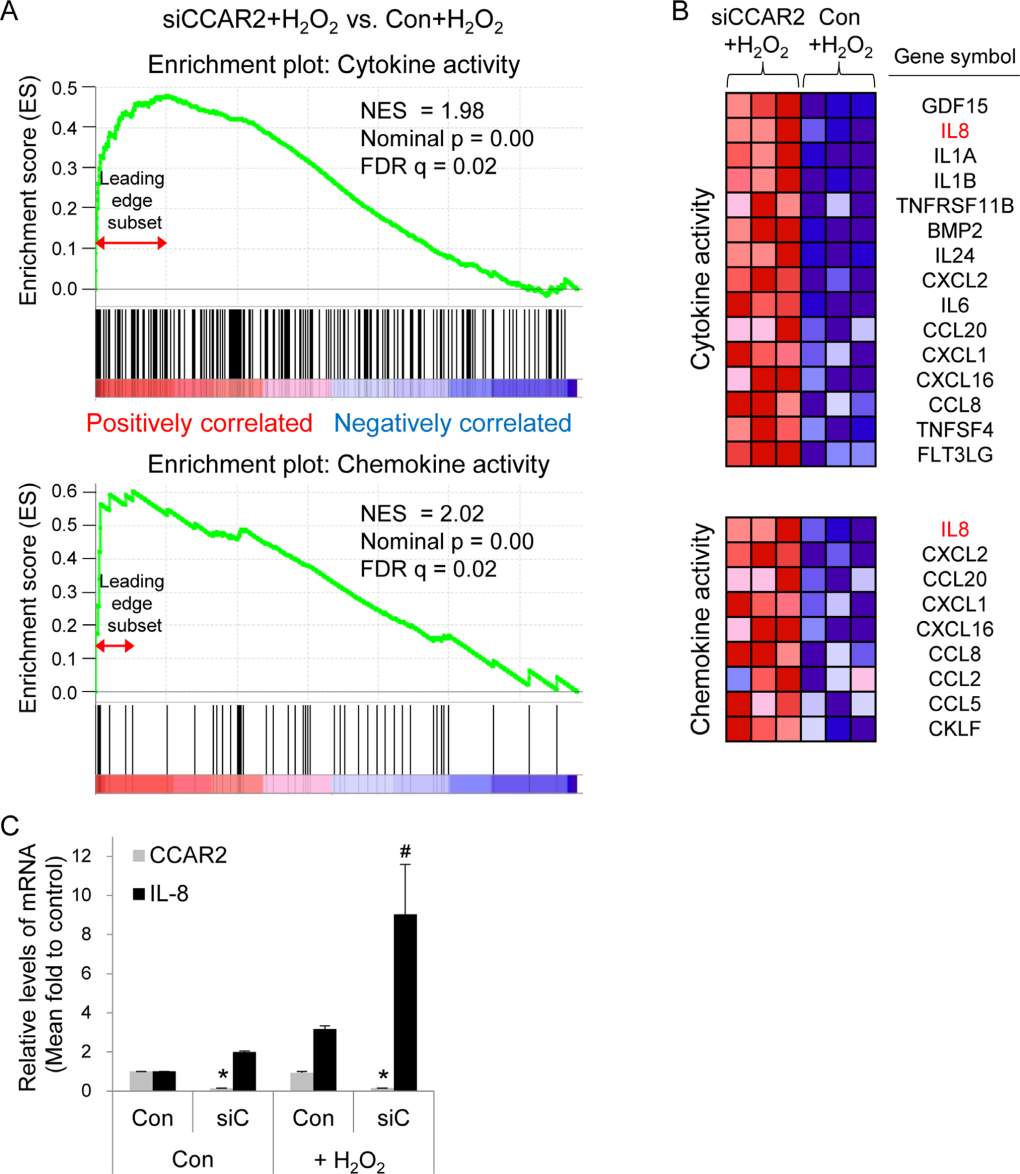
CCAR2 deficiency upregulates cytokine and chemokine following H_2_O_2_ treatment HeLa cells were transfected with siCCAR2 (siC). Forty-eight hours later, cells were treated with 0.5 mM H_2_O_2_ for 2 h. (**A**) Gene Set Enrichment Analysis (GSEA) of gene expression profile rank gene sets (cytokine and chemokine activity), according to expression values (red, upregulated; blue, downregulated). The upper part of the plot shows progression of the running enrichment score and the maximum peak therein. The lower part shows the genes in the gene set as “hits” against the ranked list of genes. Normalized enrichment score, NES; nominal *P*-value, nominal p; false discovery rate q-value, FDR q. (**B**) The corresponding heat-map shows expression values for the top subset of genes (red, upregulated; blue, downregulated). (**C**) Bar graph shows the relative expression of each mRNA from three independent microarrays. ^*^P < 0.05 (CCAR2) and ^#^*P* < 0.05 (IL-8), significantly different from control cells.

The role of CCAR2 in the oxidative tumor microenvironment is unclear. First, to explore whether CCAR2 controls responses to oxidative stress, we used a genome-wide microarray to identify transcriptional target genes coregulated by CCAR2 and hydrogen peroxide (H_2_O_2_) (Figure 1A). Differentially (2-fold change (FC) up or down) regulated genes corresponding to individual probes were counted and compared (Figure 1B). Following H_2_O_2_ treatment, 78 targets in CCAR2 siRNA-transfected (siCCAR2) cells showed differential expression when compared with non-transfected control cells (27 upregulated and 51 downregulated) (Figure 1B). Among them, seven genes corresponding to eight probes showed oxidative stress-dependent transcriptional activation: *IL1A*, *NEU4*, *FOS*, *RPRM*, *NR4A2*, *OASL*, and *IL8* (Figure 1C). The 78 differentially regulated targets in H_2_O_2_-treated siCCAR2 cells were classified according to biological process using Gene Ontology (GO) analysis to identify the molecular functions most sensitive to CCAR2 levels in response to H_2_O_2_. Genes involved in inflammatory responses were significantly affected in H_2_O_2_-treated siCCAR2 cells (Figure 1D). Next, we performed Gene Set Enrichment Analysis (GSEA) to further characterize differences between control cells and siCCAR2 cells in response to H_2_O_2_. GSEA revealed that cytokine and chemokine activity gene sets were enriched significantly among genes upregulated in H_2_O_2_-treated siCCAR2 cells (Figure 2A). This suggests that CCAR2 regulates production of cytokines and chemokines under oxidative conditions. Indeed, expression of interleukins and chemokine ligands including IL-8, IL-1, IL-24, IL-6, CC chemokines, and CXC chemokines increased significantly (Figure 2B). In particular, expression of IL-8 showed the most significant fold change in H_2_O_2_-treated siCCAR2 cells compared with control cells (Figure 2C). CCAR2 expression was not significantly altered by H_2_O_2_ treatment (Figure 2C). Overall, these data suggest that CCAR2 plays a key role in regulating H_2_O_2_-responsive genes.

### CCAR2 regulates IL-8 production

**Figure 3:**
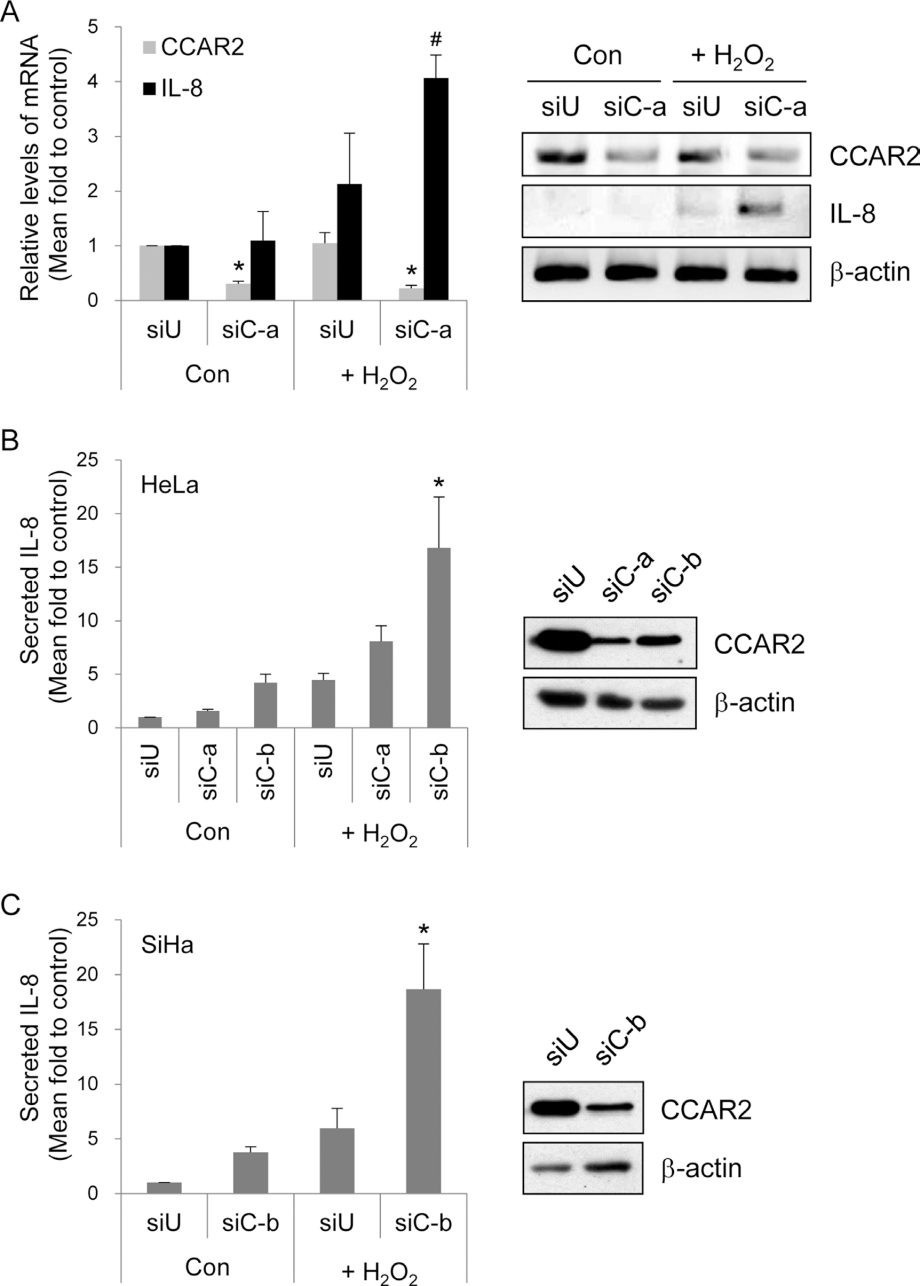
CCAR2 deficiency promotes IL-8 production following H_2_O_2_ treatment Cells were transfected with siUniversal (siU) or siCCAR2 (siC-a or siC-b). (**A**) Forty-eight hours after transfection, HeLa cells were treated with 1 mM H_2_O_2_ for 2 h. Expression of CCAR2 and IL-8 mRNA was validated by semi-quantitative RT-PCR (right panel). The band of PCR product and background on agarose gel was inverted to black and white, respectively. The levels of each mRNA were normalized to that of β-actin and are shown in the bar graph (left panel). ^*^P < 0.05 (CCAR2) and ^#^*P* < 0.05 (IL-8), significantly different from control cells. (**B**, **C**) Twelve hours after treatment of HeLa or SiHa cells with 1 mM H_2_O_2_ for 2 h, culture supernatants containing extracellular IL-8 were collected for ELISA. Intracellular levels of CCAR2 protein were confirmed by Western blotting (right panel). Relative levels of secreted IL-8 protein are shown in the bar graph (left panel). ^*^P < 0.05, significantly different from control cells.

Among cytokines and chemokines regulated by CCAR2 following oxidative stress, expression of IL-8 mRNA showed the most significant change in the microarray. Significant upregulation of IL-8 expression by H_2_O_2_-treated siCCAR2 cells was validated by RT-PCR (Figure 3A). To confirm IL-8 secretion into the extracellular space, we measured the amount of IL-8 in culture medium. IL-8 production by H_2_O_2_-treated cells was higher than that by non-treated control cells. Production of IL-8 protein was significantly induced in siCCAR2-HeLa and siCCAR2-SiHa cells than that in siUniversal-transfected cells following H_2_O_2_ treatment (Figure 3B and 3C). This demonstrates that CCAR2 deficiency potentiates IL-8 upregulation following oxidative stress. In addition, the data suggest that CCAR2 deficiency fosters a pro-inflammatory tumor microenvironment.

### The upregulation of IL-8 is mainly mediated by AP-1 transcription factor

**Figure 4:**
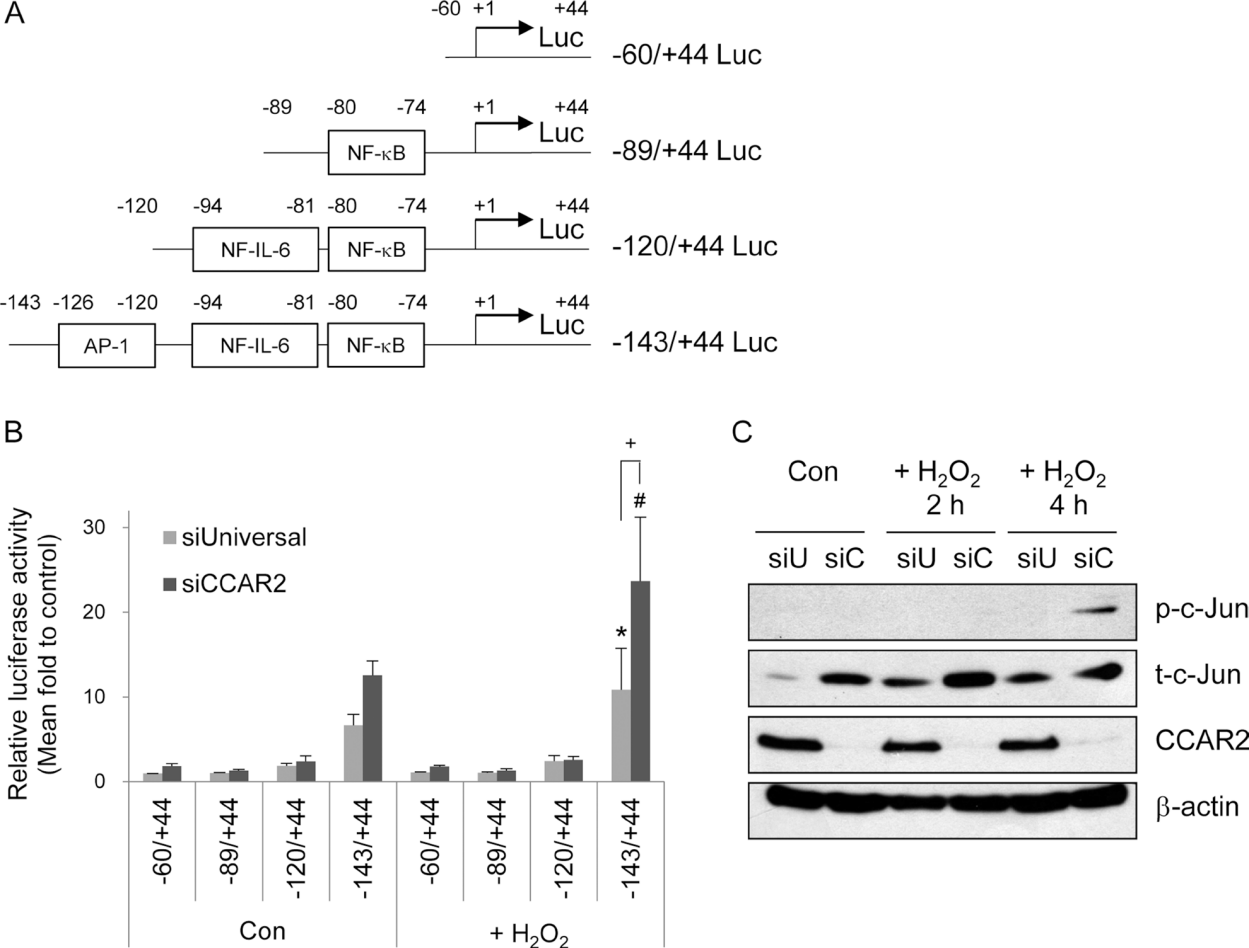
CCAR2 deficiency-induced IL-8 expression is mediated by the AP-1 transcription factor (**A**) Schematic representation of the human IL-8 promoter luciferase (Luc) constructs. (**B**) HeLa cells were transfected with siUniversal or siCCAR2 and treated with 0.5 mM H_2_O_2_ for 2 h. Relative luciferase activity was calculated after normalization of transfection efficiency according to β-galactosidase activity. ^*^P < 0.05 (siUniversal) and ^#^*P* < 0.05 (siCCAR2), significantly different from the shortest Luc reporter. ^+^*P* < 0.05, significant different between siUniversal and siCCAR2 cells. (**C**) HeLa cells were transfected with siUniversal (siU) or siCCAR2 (siC) and treated with 0.5 mM H_2_O_2_ for 2 or 4 h. Expression of each protein was analyzed by Western blotting.

To investigate the mechanism by which CCAR2 regulates IL-8 transcription, cells were transfected with luciferase reporter constructs containing the IL-8 promoter harboring binding sites for various transcription factors (AP-1, NF-IL-6, and NF-kB) (Figure 4A). Both before and after H_2_O_2_ treatment, the full length ( – 143/ + 44) promoter-containing reporter generated higher luciferase activity than the shorter promoter-containing reporters in both siUniversal and siCCAR2 cells (Figure 4B). In addition, siCCAR2 cells showed the highest luciferase activity after H_2_O_2_ treatment (Figure 4B). Furthermore, the increase in luciferase activity in siCCAR2 relative to that in siUniversal cells was significant only in cells harboring the full length reporter after H_2_O_2_ treatment (Figure 4B). This implies that the AP-1 transcription factor mediates upregulation of IL-8 in CCAR2-deficient cells under oxidative stress. AP-1 is a dimeric transcription factor comprising Jun, Fos, ATF, Maf, and JDP family members [30]. Among these, we measured activation of c-Jun by measuring its phosphorylation. siCCAR2 cells showed increased phosphorylation of c-Jun in response to H_2_O_2_ (Figure 4C). This demonstrates that AP-1 activation is required for IL-8 upregulation in CCAR2-deficient cells.

### The expression of CCAR2 and IL-8 is negatively correlated

**Figure 5:**
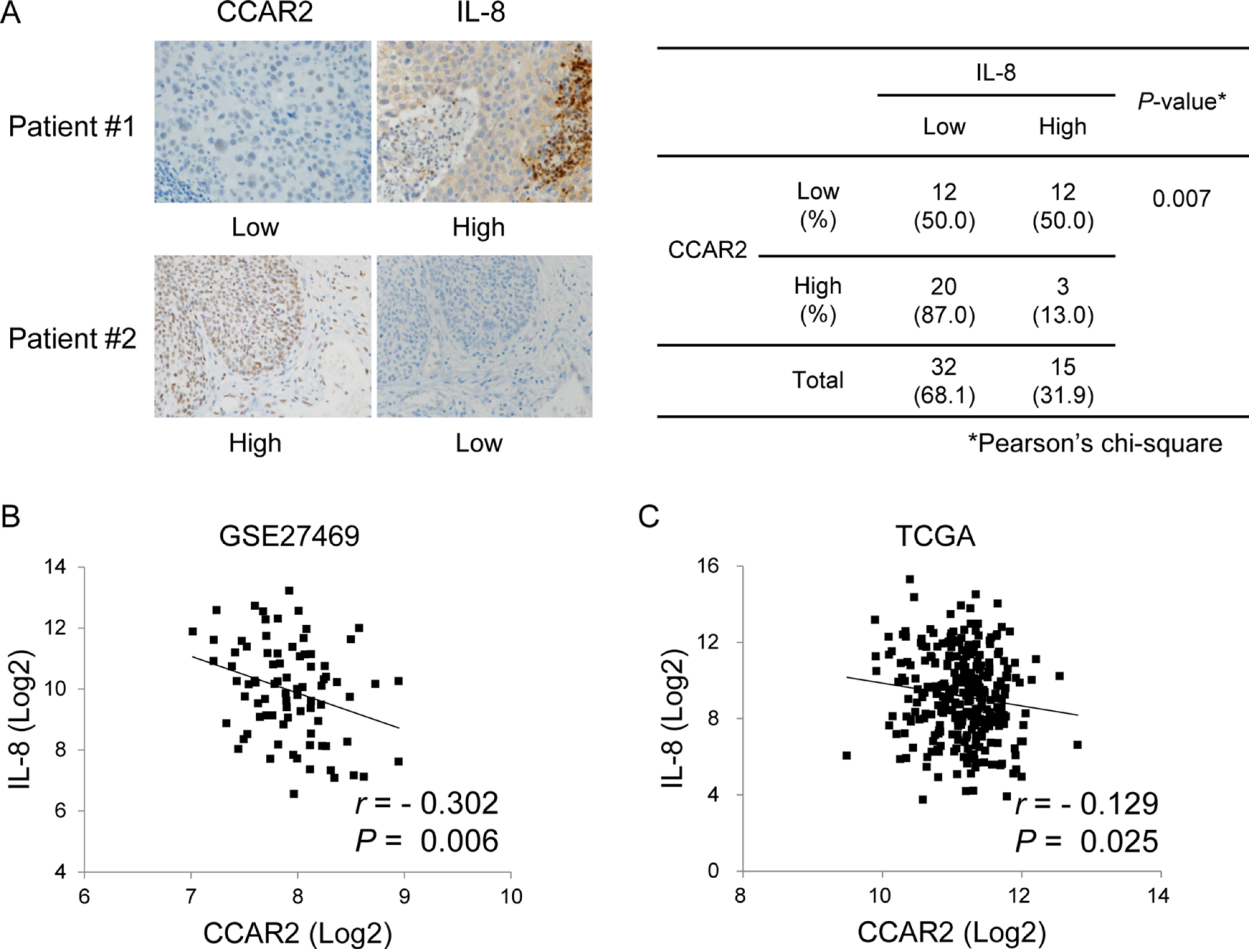
CCAR expression in tissues from cervical cancer patients is negatively correlated with that of IL-8 (**A**) Expression of CCAR2 and IL-8 protein in tumor tissues from cervical cancer patients was examined by IHC (*n* = 47). The immunostaining score is described in the Methods. The association between CCAR2 and IL-8 expression was determined using Pearson’s chi square (χ^2^) test. (**B**, **C**) Information regarding expression of CCAR2 and IL-8 mRNA in tumor tissues from cervical cancer patients was downloaded from the GEO (GSE27469) (*n* = 82) (B) and TCGA (*n* = 304) (C) databases. Pearson correlation analysis was used to quantify the relationship between CCAR2 and IL-8. *r*, Pearson’s correlation coefficient; *P*, *P*-value.

The above data demonstrate that CCAR2 is one of the factors that regulate IL-8 expression *in vitro*. Next, CCAR2-dependent IL-8 expression was verified in cervical tumor tissues isolated from cervical cancer patients (Table 1). Immunohistochemistry (IHC) revealed that, while CCAR2 expression was confined to the nucleus, that of IL-8 was evident mainly in the cytoplasm, or focally in the nucleus. The association between CCAR2 and IL-8 showed a significant negative correlation (Figure 5A). To confirm this negative correlation, we examined mRNA expression using data derived from 82 and 304 cervical cancer patients downloaded from Gene Expression Omnibus (GEO) (Figure 5B) and The Cancer Genome Atlas (TCGA) (Figure 5C), respectively. Expression of CCAR2 showed a weak (–0.40 < *r* <–0.20) (Figure 5B) and very weak (–0.20 < *r* < 0.00) (Figure 5C) negative relationship, respectively, with IL-8. These negative relationships were statistically significant (*P* < 0.05). This suggests that CCAR2 is a negative regulator of IL-8 expression.

**Table 1:** Characteristics of cervical cancer patients used for IHC

Characteristics	Number
Total patients	47
Age	
< 29	2
30 ∼ 39	6
40 ∼ 49	16
50 ∼ 59	8
60 ∼ 69	11
> 70	4
Subtypes	
Squamous Cell Carcinoma	33
Adenocarcinoma	7
Carcinoma in situ	5
Adenocarcinoma in situ	1
Small cell carcinoma	1

### High expression of IL-8 is associated with low overall survival of cervical cancer patients

**Figure 6:**
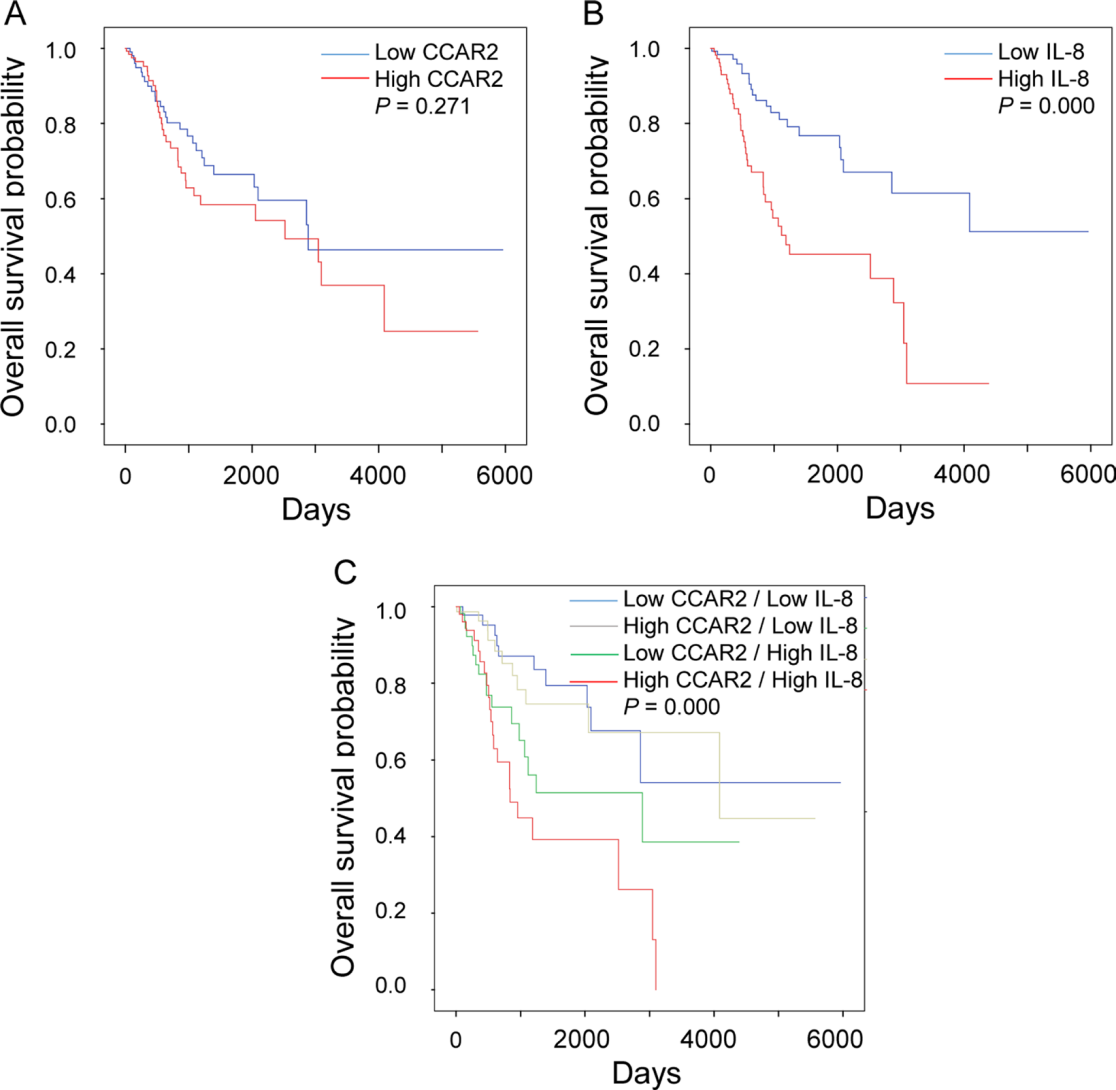
CCAR2-dependent IL-8 expression is associated with low survival of cervical cancer patients (**A**, **B** and **C**) mRNA expression data set was obtained from TCGA for cervical cancers. Expression of CCAR2 and IL-8 was defined as high (above median) or low (below median). Kaplan-Meier survival analysis was performed using data from 304 patients. *P*-values were calculated using the log-rank test.

Next, we investigated the association between expression of CCAR2 and IL-8 and overall survival. While the level of CCAR2 expression had no effect on overall survival, high expression of IL-8 is associated with shorter survival of cervical cancer patients (Figure 6A and 6B). Thus, it suggests that CCAR2 itself does not affect the survival of cancer patients, but CCAR2-dependent IL-8 expression does. Next, we divided the patients into four groups based on the expression status of each gene: (i) low CCAR2/low IL-8, (ii) high CCAR2/low IL-8, (iii) low CCAR2/high IL-8, and (iv) high CCAR2/high IL-8. As expected, overall survival of the low CCAR2/high IL-8 group was shorter than that of the low CCAR2/low IL-8 and high CCAR2/low IL-8 groups (Figure 6C), suggesting that low CCAR2 expression is associated with high IL-8 expression and contributes to create aggressive tumor microenvironment. However, unexpectedly, we found that the overall survival of the high CCAR2/high IL-8 group was shorter than that of the low CCAR2/high IL-8 group. The high CCAR2/high IL-8 group showed the poorest overall survival rate (5 year overall survival, 39%; *P* = 0.000). These data suggest that CCAR2 also plays an independent role in patients with high IL-8 expression. Overall, high IL-8 expression regulated by CCAR2 plays a critical role in aggressive progression of cervical cancer.

## DISCUSSION

Here, we showed that CCAR2 negatively controls the expression of IL-8 through the regulation of AP-1 transcription factor. Considering that IL-8 is a chemokine fertilizing tumor microenvironment, CCAR2 is one of critical factors to be tightly regulated.

IL-8 is expressed at high levels in various cancers, including breast, colorectal, gastric, ovarian, pancreatic, prostate cancer, and melanoma [25]. In addition, the high expression of IL-8 is associated with low survival of patients with basal-like breast cancer [31], colorectal cancer [32], chronic lymphocytic leukemia [33, 34], hilar cholangiocarcinoma [35], lung adenocarcinoma [31], non-small cell lung carcinoma [31], pancreatic cancer [36], and ovarian cancer [31, 37, 38]. The strong correlation between IL-8 expression and low survival is explained by the aggressive role of IL-8, which promotes mitogenesis, angiogenesis, and invasion. In addition to its tumorigenic effects, IL-8 is related to chemotherapeutic responses. Chemotherapeutic agents upregulate IL-8 expression: a characteristic of multidrug-resistant cells [39]. High levels of IL-8 lead to a poor response to chemotherapeutic agents such as 5-fluorouracil, paclitaxel, doxorubicin, cisplatin, and oxaliplatin [40–43]. Thus, IL-8 is a critical prognostic marker for cancer.

In addition to IL-8, CCAR2 can be used to assess a patient’s prognosis and response to chemotherapy. High expression of CCAR2 correlates with low overall survival or a poor prognosis for patients with breast cancer [44, 45], clear cell renal cell carcinoma [46], colon cancer [8, 47], diffuse large B cell lymphoma [48], esophageal squamous cell carcinoma [13], gastric cancer [9, 49, 50], hepatocellular carcinoma [51], laryngeal and hypopharyngeal carcinoma [52], osteosarcoma [6], ovarian carcinoma [53], and soft tissue sarcoma [54]. While there is a negative correlation between CCAR2 expression and survival for various cancers, our study shows that there is no correlation between CCAR2 and overall survival of cervical cancer patients. In addition, CCAR2 deficiency induces sensitivity to UV irradiation [55, 56], suggesting that CCAR2 confers resistance to genotoxic stress-induced chemotherapy.

Several studies show that CCAR2 affects gene expression profiles. CCAR2 deficiency downregulates expression of mitosis-regulatory genes in squamous cell carcinoma cells [5] and expression of Wnt/β-catenin target genes in colon cancer cells [8]. In addition, CCAR2 controls expression of pro-inflammatory cytokines, including IL-6 and IL-8. IL-6 levels are reduced at the early stage of adipocyte differentiation in CCAR2 shRNA-transfected cells, and in adipose tissues from CCAR2 knockout mice fed a high fat diet [57]. TALEN-mediated knockdown of CCAR2 in SW480 colon cancer cells [8] and shRNA-mediated depletion of CCAR2 from MDA-MB-231 breast cancer cells [58] result in low expression of IL-8 compared with that in wild-type cells. In contrast to previous studies, we found that CCAR2 siRNA-transfected cells show the upregulation of IL-8 in HeLa and SiHa cervical cancer cells following oxidative stress; the reason for this may be that previous studies used different cell types or stimuli. The detailed mechanism underlying the differential regulation of IL-8 by CCAR2 requires further investigation. Our study showed that AP-1 activation was required for the upregulation of IL-8 in CCAR2-deficient cells in the absence or presence of H_2_O_2_. Phosphorylation of c-Jun, an AP-1 family member, was involved in the AP-1 activation in H_2_O_2_-treated CCAR2-deficient cells, but not was detected in the absence of H_2_O_2_. It implies that other AP-1 family members including Fos, ATF, Maf, and JDP participate in its activation for the upregulation of IL-8. The mechanism by which CCAR2 deficiency activates AP-1 transcription factor needs to be investigated.

Until now, the mechanism underlying the role of CCAR2 in cytokine production was unclear. Here, we show that CCAR2 regulates IL-8 production under conditions of oxidative stress. Taken together, the results suggest that CCAR2 may be a potential therapeutic target for inflammatory diseases and cancer.

## MATERIALS AND METHODS

### Cell culture and treatment

HeLa and SiHa cervical cancer cells were maintained in Dulbecco’s modified Eagle’s medium (Welgene Inc., Korea) and Eagle’s minimum essential medium (Welgene Inc., Korea), respectively, supplemented with 10% fetal bovine serum, 100 U/ml penicillin G sodium, 100 µg/ml streptomycin sulfate, and 0.25 µg/ml amphotericin B. Cells were incubated at 37°C in a 5% CO_2_ incubator and treated with hydrogen peroxide (H_2_O_2_) for the indicated times.

### Transfection of small interfering RNA (siRNA)

Universal and CCAR2 siRNAs were synthesized by ST Pharm. Co., LTD. (Korea). The siRNA duplexes were as follows: universal siRNA (siU), AUGAACGUGAAUUGCUCAAdTdT; CCAR2 (NM_021174) siRNA-a (siC-a), CAGCUUGCAUGACUACUUUdTdT; CCAR2 siRNA-b (siC-b), CCAUAAUUCUUGCCUCUUUdTdT. Transfection of 20 nM siRNA was performed using Lipofectamine RNAiMax (Invitrogen, USA). All experiments were performed 48 h after transfection.

### Gene expression analysis

Total RNA was isolated from HeLa cells using an RNeasy Plus Micro kit (Qiagen, USA). Gene expression profiling was based on three independent experiments using an Illumina Human HT-12 v4 Expression BeadChip with 47,322 probes (Illumina, Inc. USA). The expression values for each gene were Log2-transformed and processed using upper quartile (UQ) normalization. Genes showing a significant difference in differential expression were selected with a cut-off value of a 2.0-fold change (FC) and a *P*-value < 0.05. Gene Ontology (GO) analysis was performed using The Database for Annotation, Visualization and Integrated Discovery (DAVID) 6.8 and functional annotation clustering. Gene Set Enrichment Analysis (GSEA) was performed using GSEA software (http://www.broadinstitute.org/gsea/). The data discussed in this publication have been deposited in the National Center for Biotechnology Information (NCBI) Gene Expression Omnibus (GEO) and are accessible through GEO series accession number GSE101612.

### Semi-quantitative reverse transcription-polymerase chain reaction (RT-PCR)

Total RNA was isolated using Trizol reagent (Invitrogen, USA), and cDNA was synthesized using PrimeScript™ reverse transcriptase (Takara, USA). The sequences of each forward (F) and reverse (R) primer used for PCR were as follows: CCAR2-F, CAAACATCCCACACACTTCAC; CCAR2-R, GACCTGGATCCGGCTTGGATG; IL-8-F, CTGTGTGAAGGTGCAGTTTTG; IL-8-R, CCTCTGCACCCAGTTTTCCTT; β-actin-F, GCTCGTCGTCGACAACGGCT; and β-actin-R, CAAACATGATCTGGGTCATCTTCTC. The PCR product was visualized by agarose gel electrophoresis in ethidium bromide gel. The levels of mRNA were quantified using ImageJ software.

### Preparation of crude cell extracts and Western blotting

Cells were lysed on ice for 10 min using NETN lysis buffer (100 mM NaCl, 1 mM EDTA, 20 mM Tris-HCl [pH 8.0], 0.5% Nonidet P-40, 50 mM β-glycerophosphate, 10 mM NaF, and 1 mM Na_3_VO_4_) containing a protease inhibitor cocktail (535140, Millipore, USA). After centrifugation at 100× g for 5 min, the supernatant was saved as a crude cell extract. This was boiled in Laemmli buffer and loaded onto a SDS-polyacrylamide gel [59]. Western blotting was performed according to a standard protocol. The following antibodies were used for Western blotting: c-Jun (9165), c-Jun-pS63 (2361), and β-actin (4970), all from Cell Signaling Technology (USA). Anti-CCAR2 antibody was generated as described previously [60].

### Enzyme-linked immunosorbent assay (ELISA)

Cells were treated with H_2_O_2_ for 2 h, and the medium was washed out. Twelve hours later, conditioned medium was collected and centrifuged to remove floating cells. The concentration of IL-8 protein in medium was measured using a human IL-8 ELISA kit (Komabiotech, Korea). The amount of IL-8 was normalized to that of total protein in the conditioned medium.

### Luciferase reporter assay

The transcriptional activity of the IL-8 promoter was measured in a luciferase reporter gene assay. Luciferase vectors containing various lengths of the human IL-8 promoter were kindly provided by Tae Sung Kim (Korea University) [61]. Cells were co-transfected with pGL3-IL-8 promoter-luciferase and pCMV-β-galactosidase. Forty-eight hours later, the cells were lysed with luciferase cell lysis buffer (25 mM Gly-Gly [pH 7.8], 15 mM MgSO_4_-7H_2_O, 4 mM EGTA [pH 8.0], 1% Triton X-100, and 1 mM DTT). Luciferase and β-galactosidase activity was measured using luciferin and *O*-nitrophenyl-β-D-galactopyranoside, respectively, as substrates. Transfection efficiency was normalized to β-galactosidase activity.

### Statistical analysis

All assays were repeated more than three times under independent conditions. Data were expressed as the mean ± standard error of mean (SEM). Individual expression values were expressed as fold increases above/below that of control cells, whose expression was set to 1 (with no variance [SEM = 0]) to reduce the effect of inter-experimental variations. Differences between three or more groups were evaluated by one-way analysis of variance (ANOVA), followed by a Tukey’s honest significant difference (HSD) comparison. Statistical differences were considered significant when *P* < 0.05 (indicated by *, ^#^, or ^+^).

### Patients

Tissue samples from 47 cervical cancer patients were obtained from the Korea Gynecologic Cancer Bank (KGCB) of the Infrastructure Project for Basic Science of the Ministry of Education, Science and Technology (MEST, Korea). The use of patient specimens was authorized by the Institutional Review Board of Kyung Hee University (KHSIRB-14-044(EA)) and KGCB (KGCB-2014-3). All patients provided informed consent via Yonsei University Gangnam Severance Hospital Gene Bank.

### Immunohistochemistry (IHC)

IHC was performed using a Bond Polymer Intense Detection System (Leica-Vision BioSystems, Germany) [45]. In brief, 4 μm sections of formalin-fixed, paraffin-embedded tissues were deparaffinized with Bond Dewax Solution and immersed in Bond ER solution for 30 minutes at 100°C to retrieve antigen. Endogenous peroxidase was quenched by incubation with H_2_O_2_ for 5 min at room temperature. The sections were incubated for 15 min at room temperature with appropriate primary antibodies using a Bond Intense R detection Kit (Vision BioSystems, Australia). Nuclei were counterstained with hematoxylin. One tissue section showing strong immunoreactivity in a pilot test was used as a positive control. Normal horse serum was substituted for the primary antibody as a negative control. All immunohistochemical slides were examined by three independent investigators (W Kim, BJ Noh, and J Lee) who were blinded to the clinical data. The percentage of stained tumor cells and its staining intensity were evaluated semi-quantitatively. To measure CCAR2 nuclear staining, H-scores were calculated using the following equation: H-score = (% cells with intensity of negative [0] × 0) + (% cells with intensity of [1+] × 1) + (% cells with intensity of [2+] × 2) + (% cells with intensity of [3+] × 3) [62]. The values ranged from 0 to 300. Expression was defined as high (above median) or low (below median). To measure IL-8 with cytoplasmic or nuclear staining, staining intensity was quantified using a score ranging from 0 to 3. Scores 3 and 2 were defined as high, and scores 1 and 0 were defined as low. Pearson’s chi square (χ^2^) test was conducted to measure the correlation between two proteins.

### GEO data analysis

mRNA expression data were obtained from the NCBI’s GEO database (Accession No. GSE27469). The levels of *CCAR2* and *IL-8* transcripts were obtained from 82 cervical cancer patients. After upper quartile normalization, the expression value was Log2-transformed. Pearson’s correlation coefficient (*r*) was calculated to determine the correlation between the two transcripts. The strength of correlation was interpreted following the guide of Evans [63]; 0.00-0.19, very weak; 0.20-0.39, weak; 0.40-0.59, moderate; 0.60-0.79, strong; 0.80-1.00, very strong. A *P-*value < 0.05 was considered statistically significant.

### TCGA data analysis

Gene expression data from 304 cervical squamous cell carcinoma and endocervical adenocarcinoma cases were downloaded from TCGA (https://tcga-data.nci.nih.gov). The levels of *CCAR2* and *IL-8* transcripts were determined using Illumina HiSeq2000 RNA Sequencing Version 2 (RNA-Seq V2). Normalized RSEM (RNA-Seq by Expectation-Maximization) values were processed to Log2 values. Pearson’s correlation coefficient (*r*) was calculated to determine the correlation between two transcripts. A *P-*value < 0.05 was considered statistically significant.

### Association between CCAR2 and IL-8 expression and patient survival

The RSEM values were processed as described above. Patient information, including overall survival, was downloaded from the TCGA. For each sample, expression was defined as high (above median) or low (below median). The survival time of the patients was the day of death for deceased patients or the last contact day in alive patients for censoring. The association between transcript levels and patient survival was assessed using Kaplan-Meier curves, and the significance of differences was assessed using the log-rank test. A *P-*value < 0.05 was considered statistically significant between the population survival curves.

## Abbreviations

CCAR2: cell cycle and apoptosis regulator protein 2
IL-8: interleukin-8
